# Phenotypic Drug Discovery for Human African Trypanosomiasis: A Powerful Approach

**DOI:** 10.3390/tropicalmed5010023

**Published:** 2020-02-05

**Authors:** Frederick S. Buckner, Andriy Buchynskyy, Pendem Nagendar, Donald A. Patrick, J. Robert Gillespie, Zackary Herbst, Richard R. Tidwell, Michael H. Gelb

**Affiliations:** 1 Center for Emerging and Reemerging Infectious Diseases, Department of Medicine, University of Washington, Seattle, WA 98109, USA; jrgilles@uw.edu (J.R.G.); zherbst@uw.edu (Z.H.); 2 Department of Chemistry, University of Washington, Seattle, WA 98195, USA; andriyb@uw.edu (A.B.); pendem2@uw.edu (P.N.); gelb@chem.washington.edu (M.H.G.); 3 Department of Pathology and Laboratory Medicine, University of North Carolina, Chapel Hill, NC 27599, USA; donald_patrick@med.unc.edu (D.A.P.); tidwell@med.unc.edu (R.R.T.)

**Keywords:** *Trypanosoma brucei*, human African trypanosomiasis, drug discovery, high-throughput screening, blood–brain barrier, brain permeability, pharmacology, phenotypic drug screening

## Abstract

The work began with the screening of a library of 700,000 small molecules for inhibitors of *Trypanosoma brucei* growth (a phenotypic screen). The resulting set of 1035 hit compounds was reviewed by a team of medicinal chemists, leading to the nomination of 17 chemically distinct scaffolds for further investigation. The first triage step was the assessment for brain permeability (looking for brain levels at least 20% of plasma levels) in order to optimize the chances of developing candidates for treating late-stage human African trypanosomiasis. Eleven scaffolds subsequently underwent hit-to-lead optimization using standard medicinal chemistry approaches. Over a period of six years in an academic setting, 1539 analogs to the 11 scaffolds were synthesized. Eight scaffolds were discontinued either due to insufficient improvement in antiparasitic activity (5), poor pharmacokinetic properties (2), or a slow (static) antiparasitic activity (1). Three scaffolds were optimized to the point of curing the acute and/or chronic *T. brucei* infection model in mice. The progress was accomplished without knowledge of the mechanism of action (MOA) for the compounds, although the MOA has been discovered in the interim for one compound series. Studies on the safety and toxicity of the compounds are planned to help select candidates for potential clinical development. This research demonstrates the power of the phenotypic drug discovery approach for neglected tropical diseases.

## 1. Introduction

Two *Trypanosoma brucei* subspecies, *gambiense* and *rhodesiense*, cause human African trypanosomiasis (HAT). Natural transmission occurs in 36 countries of sub-Saharan Africa via the bite of infected tse-tse flies. After the initial cutaneous inoculation, early stage (hemolymphatic) infection occurs. In the Central/West-African form (Gambian HAT), the early stage can last for months to years before progressing to late-stage infection in the central nervous system (CNS). In the East-African form (Rhodesian HAT), the early stage lasts only a few weeks to months before causing late-stage disease. Once the parasites enter the CNS, patients suffer neuropsychiatric effects that culminate in coma and death if untreated (hence the name “sleeping sickness”). Optimal treatments for HAT will address the infection in the CNS, necessitating that drugs have the ability to penetrate the blood–brain barrier (BBB). 

The therapeutic landscape for HAT has recently been upgraded with the approval of fexinidazole for the treatment of both the first-stage and second-stage of HAT due to *T. b. gambiense* in adults and children aged ≥6 years [1]. Fexinidazole represents the first all oral therapy for this disease and will likely be a major advancement over nifurtimox-eflornithine combination therapy (NECT), which requires parenteral administration (usually in a hospital setting) for the eflornithine component [2]. As much as fexinidazole is a welcome advancement, there continues to be a need for drug discovery for HAT in order to strengthen the therapeutic armamentarium: (1) for patients that cannot tolerate fexinidazole; (2) to address the inevitable risk of drug resistance; (3) to eliminate the need for a staging spinal tap before initiating therapy [3]; and (4) to respond to the unmet need of safe and effective drugs for Rhodesian HAT. Toward this end, our research group is conducting a drug discovery campaign to identify compounds that are distinct from nitroheterocycle drugs such as nifurtimox and fexinidazole, and that will meet the target product profile of an oral drug with activity for late stage HAT [4]. 

A high throughput phenotypic screen, performed at the Genomics Institute of the Novartis Research Foundation (GNF), formed the basis of this drug discovery campaign [5]. The compound library of 700,000 compounds consisted of a collection of small-molecules with druglike properties and structural diversity. The library had been previously profiled in more than 60 high throughput screens (both biochemical and cell-based), allowing for the identification and elimination of compounds with a “frequent hitter profile”. The phenotypic assay measured the inhibition of bloodstream-form *T. brucei* cultures at a single compound concentration of 3.6 µM. The screen resulted in 3889 primary hits with an inhibition of greater than 50% for a 0.6% hit rate (Z’ score > 0.6). The primary hits were further tested in dose-response assays in order to measure EC_50_ values. In parallel, the cytotoxicity (CC_50_) of the hit compounds was measured against cultures of the human hepatoma cell line (Huh7). A final set of compounds was compiled with *T. brucei* EC_50_ < 3.6 µM and CC_50_ > 10 µM, consisting of 1035 molecules that could be grouped into 115 distinct scaffolds [5]. This paper summarizes the progress to date of our drug discovery campaign to identify preclinical candidates for HAT, starting from this phenotypic screen.

## 2. Materials and Methods

The methods employed in this paper have been described in detail in previous publications as follows. All murine experiments were approved by the University of Washington Institutional Animal Care and Use Committee, IACUC approval code 4248-01 (animal welfare approval number A3464-01).

### 2.1. Phenotypic Screen for T. brucei Growth Arrest 

Compound libraries were screened against the bloodstream form of the *T. brucei* isolate, Lister 427 [5]. Parasites were grown in 1536-well plates in 5.5 μL of HMI-9 medium in the presence of library compounds. All wells including negative controls contained a final of 0.4% dimethyl sulfoxide. Plates were incubated at 37 °C for 48 h and the parasite density was determined using the CellTiter-Glo reagent (Promega, Madison, Wisconsin WI, USA), a firefly luciferase assay system that measures the amount of cellular adenosine triphosphate present in plate wells.

### 2.2. In Vitro Parasite Growth Arrest Assay 

Follow up compounds were tested for antiparasitic activity on *T. brucei brucei* (strain BF427) [5]. Parasites were tested in triplicate in the presence of serial dilutions of compound, and growth was quantified with AlamarBlue. Pentamidine isothionate (Aventis, Dagenham, U.K.) was included as a positive control in each assay (EC50 = 1.2 ± 0.3 nM).

### 2.3. Mammalian Cell Cytotoxicity Assay 

Compounds were tested for cytotoxicity against CRL-8155 cells (human lymphoblasts) [5]. Cells were grown in culture with serial dilutions of compounds for 48 h and cytotoxicity was assayed using AlamarBlue (Life Technologies, Carlsbad, California CA, USA). Each dilution was assayed in quadruplicate with the standard error of the mean values averaging < 15%. Concentrations causing 50% growth inhibition (CC50) were calculated by nonlinear regression using GraphPad Prism software (San Diego, CA, USA). 

### 2.4. Solubility Measurement 

Solubility was measured in pH 7.4, pH 6.5, and pH 2.0 aqueous buffers in a two-tier system via LC-MSMS. In Tier 1 testing, 1 μL of DMSO stock (20 mM) was measured with a Hamilton syringe and diluted to 400 μL with the respective buffer, giving a final concentration of 50 μM test compound with 0.25% DMSO. The buffer solutions were capped and incubated while shaking at 37 °C for 24 h until equilibrium was reached. Buffer solutions were centrifuged at 14,000× *g* for 15 min and two aliquots are taken from the supernatant. The concentration of the test compound in each aliquot was determined by liquid chromatography-mass spectrometry/mass-spectrometry analysis and by calculations using a linear regression of the test compound standards made over a range of known concentrations. Solubility was reported as the final concentration in the supernatant. If the concentration in the supernatant was determined to be 50 μM (maximum solubility for Tier 1), then a Tier 2 test was carried out. In Tier 2 testing, 5 μL of the test compound’s 20 mM DMSO stock was transferred to a microcentrifuge tube with a Hamilton syringe. The DMSO was then removed in a Speed-Vac concentrator and the test compound was diluted with 100 μL of the respective buffer, giving a final concentration of 1 mM test compound with negligible DMSO. The sample was heated and agitated by vortexing and by pipetting up and down to ensure the test compound was completely exposed to the buffer. The sample was then capped and incubated for 24 h while shaking at 37 °C. Buffer solutions were centrifuged at 14,000× *g* for 15 min and two aliquots were taken from the supernatant. The concentration of the test compound in the aliquots was determined by LC-MSMS, as described above. 

### 2.5. Permeability Across Monolayers of MDCKII-MDR1 Cells 

This assay utilizes Madin−Darby canine kidney cells that were transfected with the human MDR1 (P-gp) gene [5,6]. Permeability across these monolayers was measured in triplicate. The assay was performed with and without the addition of GF-120918, an inhibitor of the MDR1 efflux pump, to determine if the compound was a pump substrate. Propranolol was used as a permeable, non-MDR1 substrate control, and amprenavir was used as a permeable, MDR1 substrate control.

### 2.6. Pharmacokinetic Studies in Mice 

Test compounds were administered to mice by oral gavage followed by blood sampling at intervals of 30, 60, 120, 240, 360, 480, and 1440 min [5,7]. Compounds were dosed orally at 50 mg/kg in 0.2 mL of dosing solution (7% Tween 80, 3% ethanol, 5% DMSO, 0.9% saline). Experiments were performed with groups of three mice per compound as published. Plasma was separated and extracted with acetonitrile for measurements of the compound concentrations by liquid chromatography/tandem mass spectrometry.

### 2.7. Brain Permeability Studies 

Test compounds were injected (ip) at 5 mg/kg to three mice in a vehicle consisting of DMSO (5%), Tween 80 (7%), and EtOH (3%) in physiological saline (0.9%) solution [8]. At 1 h post injection, blood was collected, and plasma was separated by centrifugation. Simultaneously, the brain was removed and homogenized in acetonitrile. Concentrations of compound in the plasma and brain were determined via liquid chromatography/tandem mass spectrometry. Calculations of brain levels accounted for 3% volume/weight of blood in the brain. 

### 2.8. Anti-Parasite Efficacy Studies in Mice (Acute Model)

Female Swiss-Webster mice age 6−8 weeks (group size = 5) were infected with 2 × 10^4^ *T. brucei rhodesiense* STIB 900 strain on day 0, then administered the compound or vehicle for five days [5,8]. Treatments were administered orally in the same vehicle described above at a dose and schedule anticipated to maintain plasma concentrations well above the EC_50_. The first dose was 48 h after parasite injection, and dosing was 12 or 24 h apart. Parasitemia was monitored for 60 days by microscopic analysis of blood collected from tail bleeds. Cures were defined by sustained clearance of microscopic parasitemia through the end of the 60-day observation period. Mice were euthanized when parasitemia was evident on the peripheral blood slides.

### 2.9. Anti-Parasite Efficacy Studies in Mice (Chronic Model) 

Groups of six to eight mice were infected with 1 × 10^4^ *T. b. brucei* (strain TREU667) to establish a chronic infection [8]. Treatment began on day 21 post-infection, and mice received 50 mg/kg test compound orally twice per day for 10 days (total of 20 doses) in a 200 μL volume of vehicle. A control group received the vehicle with no compound and another control group received a single intraperitoneal dose of diminazene aceturate at 10 mg/kg on day 21. The diminazene aceturate clears parasites from the blood, but because it does not cross the BBB, the blood is later repopulated from parasites in the CNS. After dosing, parasitemia was monitored via microscopic examination of tail blood slides until 180 days post-infection. Mice were removed from the experiment when parasites were detected in the blood.

### 2.10. Chemical Synthesis Procedures

For compound series 1, 2, 4, and 9, the synthesis methods are in the references. The methods for the synthesis of the initial hit compounds for compound series 3, 5, 6, 7, 8, 10, and 11 are provided in the Supplementary Materials (Figures S1–S7). Compounds were purified to > 95% purity by high performance liquid chromatography (Varian Prep star system) using a reverse phase C18 semi-preparative column (YMC S5 ODS-A 20 × 100 mm column) and a solvent program of methanol/water or acetonitrile/water with 0.1% trifluoroacetic acid.

### 2.11. Metabolite Identification

A 90-min incubation in 0.5 mg/mL mouse liver microsomes preceded liquid chromatography-mass spectrometry/mass-spectrometry analysis for each compound of interest. After incubation, aliquots drawn at various time points in the incubation period were prepared for LC-MSMS analysis alongside a microsome mixture blank containing no compound. A Thermo LTQ Orbitrap Tandem Hybrid Mass Spectrometer combined with an Acquity UPLC system was used for analysis and the MS/MS data were analyzed using MZMine 2.30 software. Peaks from the blank were compared with peaks from the microsome incubation mixture aliquots taken at different timepoints in order to determine whether the detected analyte was a product of the background components or a metabolic product of the compound in question.

## 3. Results

### 3.1. Selection of Hit Compounds

The set of 1035 active/selective compounds was further curated by a committee of three medicinal chemists involved in the project. The following filters were applied, resulting in 17 selected compounds of different chemical scaffolds: (1) compliance with the Lipinski’s rule of 5 (MW < 500, Log P not > 5, not more than 5 H-bond donors, not more than 10 H-bond acceptors) [9]; (2) avoidance of compounds with structural alerts for toxicity (e.g., avoiding alkylating agents, etc.) [10]; (3) avoidance of molecules with > 1 chiral center (to help simplify synthesis and control costs of goods); (4) avoidance of singletons, which meant excluding compounds for which analogs of the same scaffold in the library were inactive as this suggested that further optimization would be difficult to accomplish; and (5) emphasis on chemical tractability as judged by the expertise of the medicinal chemists. The structures, chemical properties, and screening results of the 16 selected hit compounds are shown in Table 1a,b. 

### 3.2. Screening for Brain Permeability

The long-term goal of the project was to develop a drug for treatment of HAT including late-stage disease involving the central nervous system. For this reason, we included an early screen in our compound triage process to identify hit compounds that demonstrated at least moderate brain permeability in mice. To assess this, the sixteen hit compounds were injected subcutaneously and mice were sacrificed at 60 min (n = 3 mice) for the simultaneous collection of plasma and whole brains for the quantification of compound concentrations. The ratios of brain to plasma levels are shown in Table 1. Eleven compounds (**1–10, 12**) had brain levels of at least 25% of plasma levels and were considered candidates for development. One compound (**11**) had modest brain permeability of 9%, but was included for further chemistry despite this marginal activity. Four compounds (**13–15, 17**) had minimal concentrations in the brain (< 2% of plasma levels) and were no longer pursued. One compound (**16**) had nearly undetectable plasma and brain levels at the 60-min time point and was also considered unsuitable for further development. 

### 3.3. Hit-to-Lead Optimization

**Figure 1:**
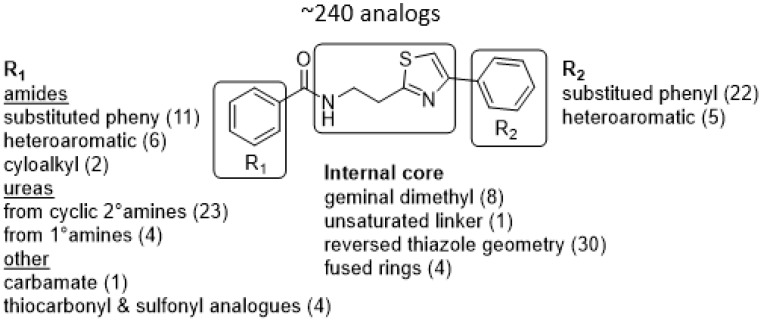
Medicinal chemistry strategy for optimization of Scaffold 2.

The program supported three medicinal chemists through a six-year period. Eleven hit compounds representing different scaffolds were pursued with a total of 1539 compounds synthesized. Lead optimization was generally performed on 2–3 scaffolds at a time due to the available manpower. Since the molecular targets or mechanisms of action were unknown for all the hit compounds at the start of the project, new compounds were designed using standard medicinal chemistry principles [12]. For illustration, compounds such as **2** were divided into regions where small changes were discretely introduced by synthetic methods (Figure 1). New compounds were made on a 3–5 milligram scale at >95% purity by HPLC, nuclear magnetic resonance, and mass spectrometry analysis.

**Figure 2:**
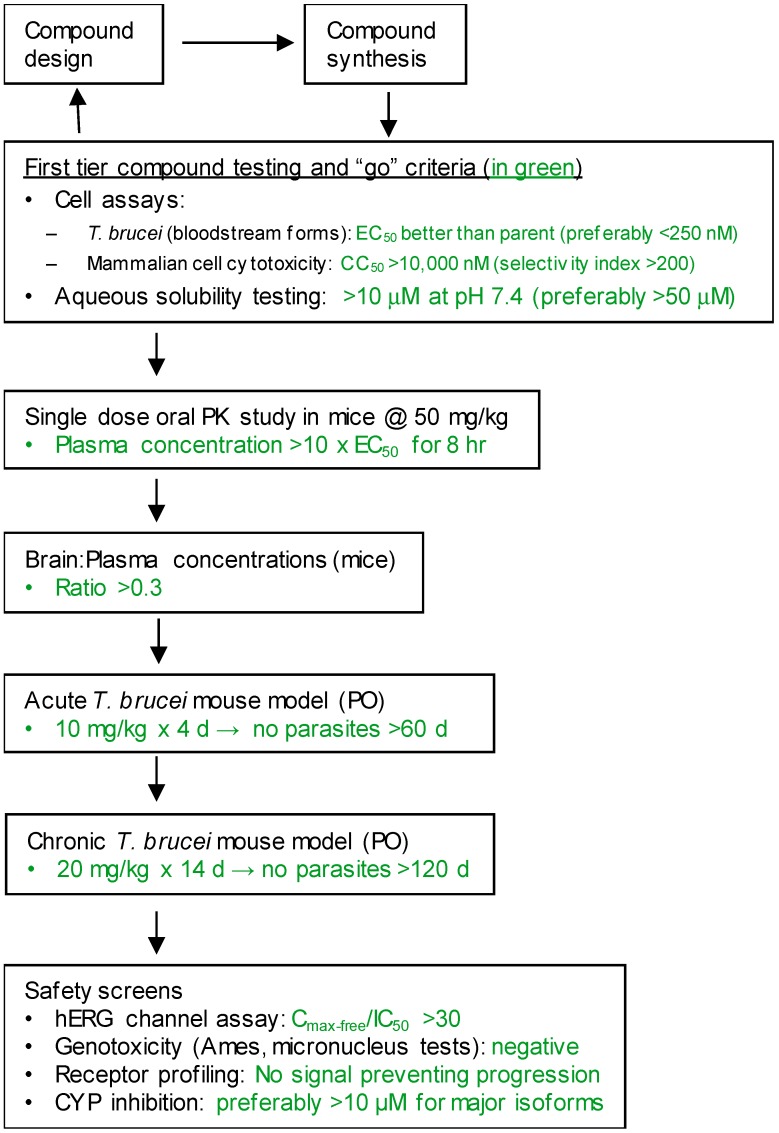
Screening cascade.

**Figure 3:**
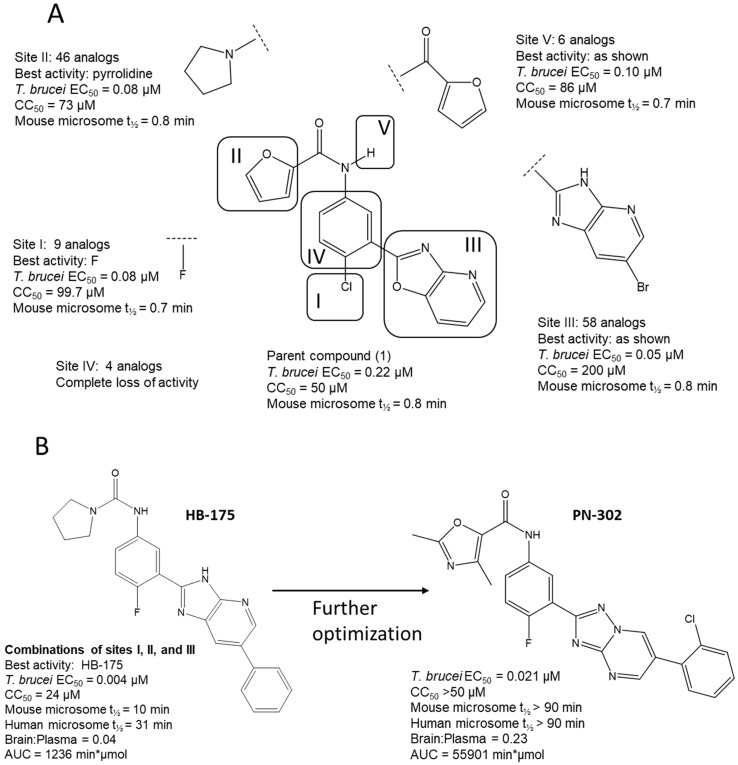
Optimization (**A**) and further optimization (**B**) of Scaffold 1.

New compounds had *T. brucei* EC_50_, mammalian host cell cytotoxicity (CC_50_), and aqueous solubility assayed per our screening cascade (Figure 2). The cut-off values for further advancement are shown. For most compound series, an initial major focus was to improve activity against *T. brucei* cells to an EC_50_ < 200 nM before concentrating on pharmacokinetic (PK) activity. The screening results were continuously reviewed by the chemistry group to define structure activity relationships (SAR). Changes in different regions of the molecule that resulted in improved activities were combined in subsequent molecules. As compounds were identified with substantially improved potency against *T. brucei* (and retained selectivity compared to mammalian cells), they were tested in a single dose PK assay in mice. Mice were administered compounds by oral gavage at 50 mg/kg and whole blood samples were collected on blotting cards at serial time points. The parameters of maximum blood concentration (C_max_) and the concentration of compound integrated over time (area under curve, AUC) were of primary interest. The general rule was to attain plasma concentrations 10 times above the *T. brucei* EC_50_ value for at least eight hours. For some series, we performed in vitro microsome stability assays prior to the PK experiments (Figure 3), although our experience was that microsome studies were just as expensive and labor intensive as “shotgun” PK experiments and provided less information, particularly about oral bioavailability. In order to improve the PK profile of compounds, substitutions were introduced that were associated with improved metabolic stability such as introducing fluorine groups to protect possible oxidation sites or introduction of N-cycloalkyl groups (pyrrolidine, piperidine) to decrease oxidative N-demethylation [12,13]. Selected compounds were subjected to metabolite identification studies to define metabolic weak points to inform the design of the next round of compounds to improve the metabolic profile (see Methods). Compounds that matched the selection criteria for antiparasitic activity and PK properties were next subjected to brain permeability studies in mice according to the flow chart (Figure 2). A brain-to-plasma ratio of 0.3 was the minimum value as a go/no-go requirement for further advancement. The compounds passing all of the above testing criteria were upscaled (75 mg synthesis) for efficacy studies in the *T. brucei* acute infection model in mice. Compounds showing cures in the acute infection model were then tested in the more challenging chronic infection model that requires clearing parasites from the CNS. Three compound series remained active in our program, having passed the different levels of our screening cascade (discussed below).

When roadblocks prevented progress for specific scaffolds, they were discontinued and replaced with new scaffolds from the list of 17 candidates in Table 1. The reasons for discontinuing various compound series are summarized in Table 2. One candidate scaffold (12) has yet to be pursued. 

### 3.4. Active Compound Series (Highlights)

Three scaffolds (1, 2, and 9) have been developed to the level of lead compounds ready for late preclinical studies [5,8,11,14,15,16]. The optimization of hit compound 1 is shown in Figure 3. The different regions of the molecule (I-V) are indicated in the center structure. The number of variants made and tested, and the optimal substitution at each region are indicated in the surrounding structures. At the bottom left (Figure 3), the partially optimized compound (HB-175) is shown, which combines the best substituents of regions I, II, and III [5]. The changes included efforts to improve metabolic stability by making the following modifications: (a) replacing the furan amide with mono or di-substituted fluoro pyrrolidine ureas, or with dimethyloxazole amide; (b) by substitution at C6 of the pyridine/pyrimidine ring; and (c) replacing azabenzofurans with imidazopyridines. Subsequent work was dedicated to further improve metabolic stability and brain permeability, leading to the current lead compound PN-302. The changes included altering the core ring system from an imidazopyridine to a triazolopyrimidine [14].

**Figure 4:**
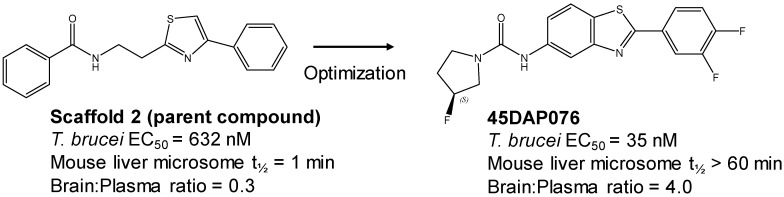
Scaffold 2: Optimization of hit “thiazole” compound to lead compound 45DAP076.

The strategy for optimizing Scaffold 2 is illustrated in Figure 1. Changes that improved antiparasitic activity, metabolic stability, and brain permeability included: (1) substitution of the phenyl group on the left side with 3-fluoropyrrolidine; (2) rigidifying the linker with a benzthiazole as opposed to an alkyl-linked thiazole; and (3) fluorination of the right-sided phenyl group. Of note, the stereochemistry of the fluorine substituent on the pyrrolidine was critical for activity [8]. The illustrated lead compound, 45DAP076, has excellent metabolic stability and excellent brain permeability properties (Figure 4). Importantly, it was shown to have *curative* activity in the chronic *T. brucei* infection model [8], putting it in a category of very few compounds with such high potential for development for HAT.

**Figure 5:**
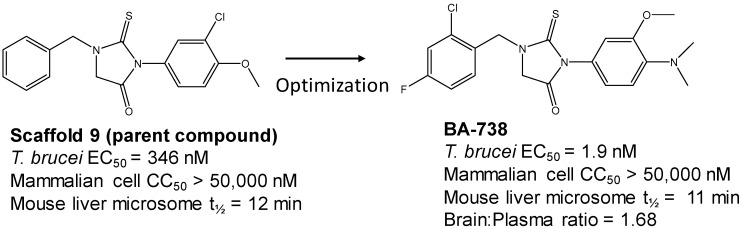
Scaffold 9: Optimization of hit “thiohydantoin” compound to lead compound BA-738.

The third compound series that remained active in the program was Scaffold 9, the thiohydantoins (Figure 5). Changes to the central thiohydantoin moiety itself abrogated antiparasitic activity, so this portion of the molecule was held constant. However, through making systematic substitutions in the two terminal rings systems, we identified highly potent inhibitors (EC_50_ as low as 2 nM) and excellent brain permeability (brain:plasma = 1.68). Compound BA-738 cured mice with acute *T. brucei* infection [11], but only gave partial cures (20% of mice) in the chronic infection model (unpublished). Further optimization will be necessary before advancing this series for late-preclinical studies such as the safety screens shown in Figure 2.

## 4. Discussion

This paper summarizes the results of a drug discovery campaign to identify preclinical candidates for HAT starting from a phenotypic screen. Detailed results relating to four of the scaffolds have been published (see references in Table 2), but an overview of the general strategy and complete results has not been previously reported. Some helpful points can be learned by studying the failed scaffolds as well as by studying the successful ones. As indicated, 17 compounds representing distinct scaffolds (Table 1) were selected from the original hit list of 1035 compounds. By definition, these compounds had activity against *T. brucei* cells and thus demonstrated sufficient cell permeability to reach intracellular targets. This feature of the cell-based screen provided a theoretical advantage over biochemical (acellular) screens where hit compounds subsequently have to be tested for (and perhaps optimized for) cell permeability properties. Similarly, the screening protocol included a counter screen against mammalian cells that eliminated compounds with cytotoxicity. A whole-cell cytotoxicity assay identified any type of cellular toxicity and thus was broader than a counter screen against, for example, a mammalian homolog in an enzymatic screen. Thus, these features illustrate the potential advantages of phenotypic screens over target-based screening with the acknowledged disadvantage that the target of activity is unknown. As a result, the hit-to-lead optimization was done agnostically to the target. For most of the project, we chose not to divert time and resources to the effort of target identification. Hit-to-lead chemical optimization was done using standard medicinal chemistry approaches without guidance from protein crystal structures. The target of compound series **1** (the trypanosome proteasome) has subsequently been identified [18], but this did not contribute to designing or synthesizing the current lead compounds (Figure 3). The results of the program to-date are that three of 11 scaffolds (27%) have been optimized to the point of giving cures in the murine model of *T. brucei* infection. More work needs to be done before compounds are brought to clinical trials, but the output of this campaign shows strong promise for delivering clinical candidates, particularly when compared to the results of target-based screening efforts for other microbial pathogens. For example, GlaxoSmithKline reported the outcome of 70 high-throughput screening campaigns (67 target based and three whole cell) for antibiotic development with only five leads delivered, translating to a 7% success rate [19]. 

As mentioned, the target product profile for HAT dictates that the final drug be administered orally [4], thus we filtered the hit list for compounds that were compliant with Lipinski’s rule of five [9]. There were some examples (e.g., compounds 14 and 15) for which the rules were slightly relaxed, although this proved to be disadvantageous as those compounds were terminated due to poor brain permeability. The decision to triage compounds early in the campaign based on brain permeability was done for the following reasons. First, it has been reported that 98% of small molecule drugs do not cross through the BBB [20], meaning the BBB is a major obstacle for developing drugs intended for CNS diseases. Furthermore, the predictive tools to design changes in molecules to improve brain permeability are unreliable when applied to diverse sets of compounds. Thus, we reasoned that it would be helpful to identify compounds with at least moderate brain permeability properties at the start, rather than struggling to try to build in this property later in the process. In order to improve the probability of brain penetrant compounds, we favored molecules with MW < 450 as this has been shown to be an approximate cut off for BBB permeability [21]. A caveat to the brain permeability studies was that the measurements were of total, rather than free, concentrations of the compounds. Thus, it is possible that some compounds were concentrated in the brain due to high tissue binding (e.g., from lipophilicity), and could be inaccessible to bind targets in the trypanosomes. With only one exception (compound **7** with MW of 469 g/mol), all the compounds that passed the permeability test of a brain to plasma ratio of > 0.25 had MWs < 450 g/mol (Table 1). In contrast, the remaining compounds that failed the permeability test had a MW > 450 g/mol with the exception of **12**, which had a MW of 391 g/mol. Amongst the 11 scaffolds that were further developed, only one (compound series **7**, which had the highest starting MW) failed to advance due to the inability to maintain or improve adequate brain permeability. The results indicate that the strategy to select compounds for good brain permeability early in the process was effective. 

As noted above, the synthesis of new compounds was not guided by structure-based drug design. Rather, design and synthesis were guided by standard medicinal chemistry approaches [12]. Specifically, hit molecules were divided into specific regions and substitutions were systematically introduced (Figure 1) to provide analogs for biological testing as per our screening cascade (Figure 2). The results of the biological testing were returned to our chemistry group to generate SAR that informed iterative rounds of synthesis and optimization. As regions of the scaffolds were improved, the various substitutions were combined, often leading to additive or multiplicative improvements. The pharmacological properties of the compounds were evaluated early in the screening process given the importance of optimizing this parameter. The “shotgun PK” experiments provided C_max_ and AUC values for initial insights into the absorption, distribution, metabolism, and elimination (ADME) of the compounds. Some scaffolds were also analyzed in microsome stability assays to help track the rates of metabolic degradation by CYP450 enzymes [8,11]. When typical methods such as fluorination did not adequately help with metabolic stability, we performed metabolite identification studies with qualitative mass spectrometry (using incubations of compound in liver microsomes) to understand the molecular targets of degradation so that new analogs could be designed to overcome the weaknesses. In rapid succession, the compounds matching our “go” criteria were then tested for in vivo CNS permeability in mice. At an early stage in the program, we utilized an in vitro trans-well methodology using MDR1-MDCK cells [5] to model permeability across the BBB [6]. Although this method has been widely used, we had specific examples in which the results of the MDR1-MDCK assay were not consistent with the in vivo brain-permeability data (not shown). We also determined that in vivo brain-permeability studies could be efficiently performed with three mice per compound at a single harvest time of 60 min post-dose (5 mg/kg IP). The combination of fewer specimens for mass spectrometry analysis plus greater predictive accuracy made the in vivo experiments preferable to the MDR1-MDCK model in our view.

The reasons for scaffold failures are indicated in Table 2. The most common reason for discontinuation (five scaffolds) was failure to make significant improvement in anti-*T. brucei* activity (EC_50_). There were no absolute criteria, but if EC_50_ values of < 200 nM could not be achieved, a compound series was stopped. The number of compounds made in these failed series were 41, 47, 66, 91, and 131, respectively. Obviously, it was a judgement decision as to when to no longer expend resources on a scaffold due to failure to achieve the sufficient target efficacy, but the listed scaffolds that failed typically involved the work of one chemist over a period of approximately one year. The next most common reason for failure (Scaffolds 5 and 7) involved difficulties achieving the desired PK endpoints. This primarily involved the failure to achieve robust plasma exposure of the compounds so that in vivo antiparasitic activity was unlikely to be achievable. The underlying problem with Scaffold 5 was presumably related to poor aqueous solubility (< 1 µM at pH 7.4 and pH 2.0). For Scaffold 7, it was poor metabolic stability that precluded its further development. Finally, Scaffold 4 was discontinued because of a “static” killing mechanism [17]. This became apparent during in vivo efficacy experiments when administration of the compound resulted in temporary suppression of parasites followed by rebound. The “static mechanism could be recapitulated in vitro in “washout” experiments [17], which was latter incorporated into our screening routine to avoid repeating the problem. 

As mentioned, the biochemical targets of the compounds were unknown at the start of the program. Work through collaborators at Genomics Institute of the Novartis Research Foundation (with contributions from the University of Washington group) led to the discovery of the target of Scaffold 1 as the trypanosome proteasome [18]. Research against the closely related *Leishmania* parasites further confirmed that proteasome inhibition was the mechanism of action for this compound series [22]. At this more advanced stage in the program, more resources will now be committed for target identification of the remaining two scaffolds. This work could help with further optimization toward inhibiting the parasite target, for example, by allowing us to develop an enzyme assay or to obtain a crystal structure, but more importantly, the information may be useful for guiding future safety studies on the compound. If the parasite target is identified and it has human orthologs, then directed efforts can be made to optimize compounds that avoid or minimize activity on the human orthologs. The information could also guide future animal studies (and clinical trials) to help predict potential toxicities in mammalian hosts. 

## 5. Conclusions

Three compound series stemming from a high-throughput phenotypic screen remained viable in this program for HAT drug development. The lead compounds demonstrated curative activity in murine models of *T. brucei* infection. The compounds are now undergoing safety studies as indicated in the bottom of the screening cascade (Figure 2). Dose/response studies in mice are also underway to establish the optimal doses and dosing schedules that will define the pharmacodynamic parameters of the leads. Subsequent investigations will include rat toxicity studies to determine the toxicities resulting from high doses of compounds. For all three series, back up compounds are available in case we encounter significant problems with toxicity or other setbacks. The described drug discovery campaign, conducted in academic centers, remains on track for producing at least one or more late preclinical candidates for HAT.

## Figures and Tables

**Table 1:** a. Structures, properties, and screening results of the 17 selected hit compounds. b. Structures, properties, and screening results of the 17 selected hit compounds.

Scaffold	Structure	MW (g/mol)	Clog P	*T. brucei* EC50 (nM)	Mammalian CC50 (nM) *	B/P Ratio (Mouse) **
a.						
1	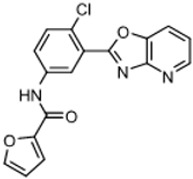	339.7	3.21	217.6	>50,000	0.547
2	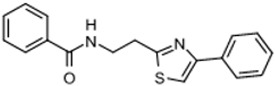	308.4	3.77	480	>50,000	0.3
3	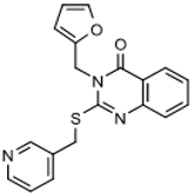	349.4	3.56	873.7	37,968	2.86
4	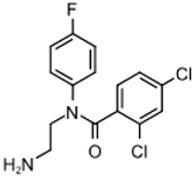	363.6	3.48	790	15,860	2.00
5	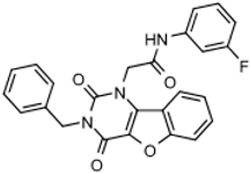	443.4	3.69	657.5	>100,000	0.845
6	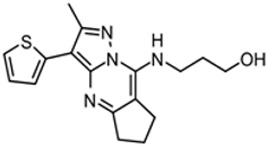	328.4	2.02	306.9	>100,000	0.727
7	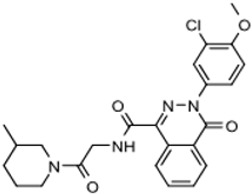	468.9	2.87	656.6	>50,000	0.587
8	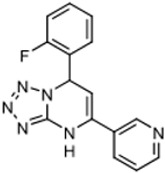	294.3	2.4	214.4	14,300	7.11
9	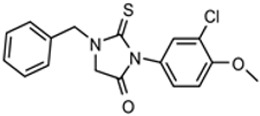	346.8	3.72	346	>50,000	0.743 ***
b.						
10	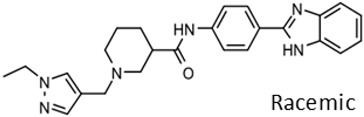	428.5	3.67	2438		0.224
11	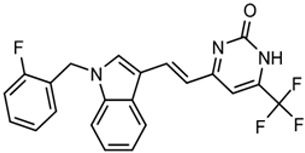	413.376	5.11	3089		0.09
12	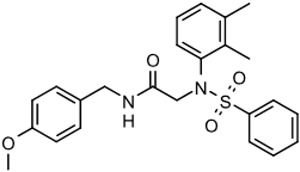	438.54	4.17	1700	>100,000	0.267
13	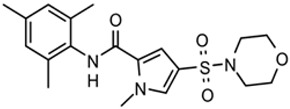	391.49	2.66	1000	>100,000	0.02
14	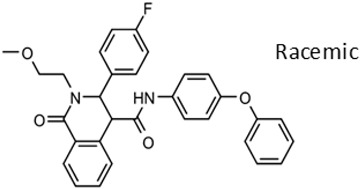	510.565	5.5	707.5	>100,000	0.01
15	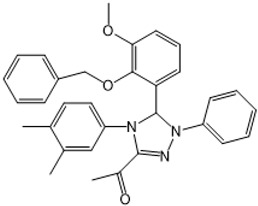	505.618	8.15	1500	33,000	0
16	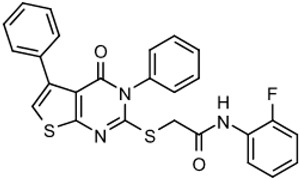	487.57	6.37	650	>100,000	No plasma exposure
17	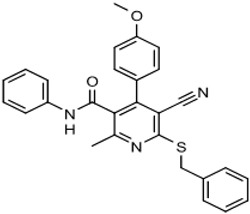	465.57	6.17	3500	>100,000	0

* Mammalian cell cytotoxicity done with lymphocytic cell line CRL-8155. ** B/P ratio = brain to plasma ratio at 60 min following IP dose (except Scaffold 8 at 15 min). *** Scaffold 9 parent had poor ionization in MS. B: P ratio data are for close analog (see [11]).

**Table 2:** Summary of progress in hit-to-lead campaigns for the 12 compound series.

Compound Series	# Analogs Made	Status	Reason for Discontinuation	Reference
1	253	Active		[5,14]
2	249	Active		[8,15,16]
3	131	Stopped	Insufficient improvement in EC50	
4	141	Stopped	Slow killing activity (“static”)	[17]
5	102	Stopped	Poor solubility. Poor PK.	
6	41	Stopped	Insufficient improvement in EC50	
7	138	Stopped	Poor metabolic stability; Poor PK	
8	91	Stopped	Insufficient improvement in EC50	
9	280	Active		[11]
10	66	Stopped	Insufficient improvement in EC50	
11	47	Stopped	Insufficient improvement in EC50	
12	0	Not started		
**Total**	**1539**			

## Supplementary Materials

The following are available online at https://www.mdpi.com/2414-6366/5/1/23/s1, Synthetic Schemes and Procedures for the Synthesis of Compound Series 3, 5, 6, 7, 8, 10, and 11. Figure S1: Synthetic scheme for compound series 3. Figure S2: Synthetic scheme for compound series 5. Figure S3: Synthetic scheme for compound series 6. Figure S4: Synthetic scheme for compound series 7. Figure S5: Synthetic scheme for compound series 8. Figure S6: Synthetic scheme for compound series 10. Figure S7: Synthetic scheme for compound series 11.

## References

[1] Deeks E.D. (2019). Fexinidazole: First global approval. Drugs.

[2] Lutje V., Seixas J., Kennedy A. (2013). Chemotherapy for second-stage Human African trypanosomiasis. Cochrane Database Syst. Rev..

[3] WHO Interim Guidelines for the Treatment of Gambiense Human African Trypanosomiasis. https://apps.who.int/iris/handle/10665/326178.

[4] DNDi HAT Target Product Profile. https://www.dndi.org/diseases-projects/hat/hat-target-product-profile/.

[5] Tatipaka H.B., Gillespie J.R., Chatterjee A.K., Norcross N.R., Hulverson M.A., Ranade R.M., Nagendar P., Creason S.A., McQueen J., Duster N.A. (2014). Substituted 2-phenylimidazopyridines: A new class of drug leads for human African trypanosomiasis. J. Med. Chem..

[6] Evers R., Cnubben N.H., Wijnholds J., Van Deemter L., Van Bladeren P.J., Borst P. (1997). Transport of glutathione prostaglandin A conjugates by the multidrug resistance protein 1. FEBS Lett..

[7] Suryadevara P.K., Olepu S., Lockman J.W., Ohkanda J., Karimi M., Verlinde C.L., Kraus J.M., Schoepe J., Van Voorhis W.C., Hamilton A.D. (2009). Structurally simple inhibitors of lanosterol 14alpha-demethylase are efficacious in a rodent model of acute Chagas disease. J. Med. Chem..

[8] Patrick D.A., Gillespie J.R., McQueen J., Hulverson M.A., Ranade R.M., Creason S.A., Herbst Z.M., Gelb M.H., Buckner F.S., Tidwell R.R. (2017). Urea derivatives of 2-aryl-benzothiazol-5-amines: A new class of potential drugs for human African trypanosomiasis. J. Med. Chem..

[9] Lipinski C.A., Lombardo F., Dominy B.W., Feeney P.J. (2001). Experimental and computational approaches to estimate solubility and permeability in drug discovery and development settings. Adv. Drug Deliv. Rev..

[10] Macherey A.C., Dansette P., Wermuth C.G. (2003). Chemical mechanisms of toxicity: Basic knowledge for designing safer drugs. The Practice of Medicinal Chemistry.

[11] Buchynskyy A.G., Gillespie J.R., Herbst Z., Ranade R., Buckner F.S., Gelb M.H. (2017). 1-Benzyl-3-aryl-2-thiohydantoin derivatives as anti-*Trypanosoma brucei* agents: SAR and in-vivo efficacy. ACS Med. Chem. Lett..

[12] Wermuth C.G. (2003). The Practice of Medicinal Chemistry.

[13] Muller K., Faeh C., Diederich F. (2007). Fluorine in pharmaceuticals: Looking beyond intuition. Science.

[14] Nagendar P., Gillespie J.R., Herbst Z.M., Ranade R.M., Molasky N.M.R., Faghih O., Turner R.M., Gelb M.H., Buckner F.S. (2018). Triazolopyrimidines and imidazopyridines as antitrypanosomal agents: Structure-activity relationships and in vivo efficacy. ACS Med. Chem. Lett..

[15] Patrick D.A., Wenzler T., Yang S., Weiser P.T., Wang M.Z., Brun R., Tidwell R.R. (2016). Synthesis of novel amide and urea derivatives of thiazol-2-ethylamines and their activity against *Trypanosoma brucei rhodesiense*. Bioorg. Med. Chem..

[16] Silva D.G., Gillespie J.R., Ranade R.M., Herbst Z.M., Nguyen U.T.T., Buckner F.S., Montanari C.A., Gelb M.H. (2017). New class of antitrypanosomal agents based on imidazopyridines. ACS Med. Chem. Lett..

[17] Buchynskyy A., Gillespie J.R., Hulverson M.A., McQueen J., Creason S.A., Ranade R.M., Duster N.A., Gelb M.H., Buckner F.S. (2017). Discovery of *N*-(2-aminoethyl)-*N*-benzyloxyphenyl benzamides: New potent *Trypanosoma brucei* inhibitors. Bioorg. Med. Chem..

[18] Khare S., Nagle A.S., Biggart A., Lai Y.H., Liang F., Davis L.C., Barnes S.W., Mathison C.J., Myburgh E., Gao M.Y. (2016). Proteasome inhibition for treatment of leishmaniasis, Chagas disease and sleeping sickness. Nature.

[19] Payne D.J., Gwynn M.N., Holmes D.J., Pompliano D.L. (2007). Drugs for bad bugs: Confronting the challenges of antibacterial discovery. Nat. Rev. Drug Discov..

[20] Pardridge W.M. (2005). The blood-brain barrier: Bottleneck in brain drug development. NeuroRx.

[21] Van de Waterbeemd H., Camenisch G., Folkers G., Chretien J.R., Raevsky O.A. (1998). Estimation of blood-brain barrier crossing of drugs using molecular size and shape, and H-bonding descriptors. J. Drug Target..

[22] Wyllie S., Brand S., Thomas M., De Rycker M., Chung C.W., Pena I., Bingham R.P., Bueren-Calabuig J.A., Cantizani J., Cebrian D. (2019). Preclinical candidate for the treatment of visceral leishmaniasis that acts through proteasome inhibition. Proc. Natl. Acad. Sci. USA.

