# Supplementary material for: Phenotypic Drug Discovery for Human African Trypanosomiasis: A Powerful Approach
Source: Trop Med Infect Dis. 2020 Feb 5;5(1):23. doi: 10.3390/tropicalmed5010023 (PMC7157241; doi:10.3390/tropicalmed5010023)
Supplement: Supplementary file 1 [file tropicalmed-05-00023-s001.pdf]

Tropical Medicine and Infectious Disease

MDPI

Article

Phenotypic Drug Discovery for Human African Trypanosomiasis: A Powerful Approach

Frederick S. Buckner [1,*], Andriy Buchynskyy [2], Pendem Nagendar [2], Donald A. Patrick [3], J. Robert Gillespie [1], Zackary Herbst [1], Richard R. Tidwell [3] and Michael H. Gelb [2]

1 Center for Emerging and Reemerging Infectious Diseases, Department of Medicine, University of Washington, Seattle, WA 98109, USA; jrgilles@uw.edu (J.R.G.); zherbst@uw.edu (Z.H.)
2 Department of Chemistry, University of Washington, Seattle, WA 98195, USA; andriyb@uw.edu (A.B.); pendem2@uw.edu (P.N.); gelb@chem.washington.edu (M.H.G.)
3 Department of Pathology and Laboratory Medicine, University of North Carolina, Chapel Hill, NC 27599, USA; donald_patrick@med.unc.edu (D.A.P.); tidwell@med.unc.edu (R.R.T.)
* Correspondence: fbuckner@uw.edu; Tel.: +1-206-616-9214 (F.B.)

Received: December 31, 2019; Accepted: 02 February 2020; Published: date

Synthetic Schemes and Procedures for the Synthesis of Compound Series 3, 5, 6, 7, 8, 10, and 11.

Compounds Series 3:

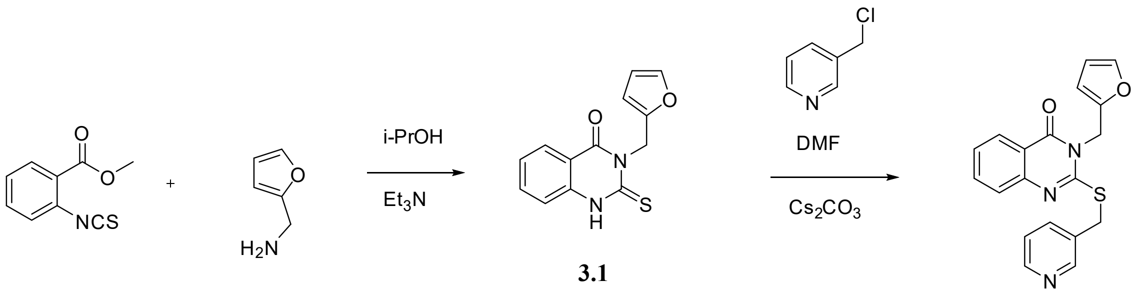

Figure S1. Synthetic scheme for compound series 3.

3.1. -[(Furan-2-Yl) Methyl]-2-Sulfanylidene-1,2,3,4-Tetrahydroquinazolin-4-One

Methyl 2-isothiocyanatobenzoate 9.65 g (50 mmol) was dissolved in 50 mL of isopropanol and to this solution 4.84 g (50 mmol) of 2-furfurylamine and 5.05 g (50 mmol) of trimethylamine were added. The resulting solution was stirred at r.t. for 2 h. The formed precipitate was filtrated, washed with isopropanol, and dried in vacuum. Obtained 11.84 g (91%) of compound **3.1** as white solid.

^{1}H NMR (DMSO-d_6) δ (ppm): 7.96 (dd, 1H, J = 1.2 Hz, J = 7.9 Hz), 7.75 (ddd, 1H, J = 1.5 Hz, J = 7.8 Hz, J = 9.0 Hz), 7.54 (dd, 1H, J = 0.6 Hz, J = 1.5 Hz), 7.39 (d, 1H, J = 8.1 Hz), 7.33 (ddd, 1H, J = 1.2 Hz, J = 7.2 Hz, J = 9.2 Hz), 6.39–6.34 (m, 2H), 5.65 (s, 2H).

3.2. -[(Furan-2-Yl) Methyl]-2-{[(Pyridin-3-Yl) Methyl] Sulfanyl}-3,4-Dihydroquinazolin-4-One

A sample of 52 mg (0.2 mmol) of compound **3.1**, 33 mg (0.2 mmol) of 3-picolyl chloride hydrochloride dissolved in 2 mL of dry DMF, and 260 mg (0.8 mmol) of cesium carbonate was added. The resulting reaction mixture stirred at 80 °C for 2 h. Progress of the reaction was monitored by TLC (10% EtOAc in $CHCl_3$). The reaction mixture was poured into 15 mL of water and the precipitate was collected, washed with water, and recrystallized from ethanol. Obtained 68 mg (97%) of pure product **3.2** as a white solid.

^{1}H NMR (300 MHz, DMSO-d$_6$) δ (ppm): 8.74 (d, 1H, J = 1.8 Hz), 8.43 (dd, 1H, J = 1.2 Hz, J = 4.5 Hz), 8.08 (dd, 1H, J = 0.9 Hz, J = 9.9 Hz), 7.92 (dd, 1H, J = 1.5 Hz, J = 9.2 Hz), 7.82 (ddd, 1H, J = 1.5 Hz, J = 7.8Hz, J = 9.1 Hz), 7.66 (d, 1H, J = 9.0 Hz), 7.58 (d, 1H, J = 0.6 Hz), 7.47 (t, 1H, J = 9.0 Hz), 7.34 (dd, 1H, J = 5.0 Hz, J = 8.1 Hz), 6.45–6.35 (m, 2H), 5.27 (s, 2H), 4.57 (s, 2H). ESI MS m/z = 350.3 ([M + H]$^+$)

Compounds Series 5:

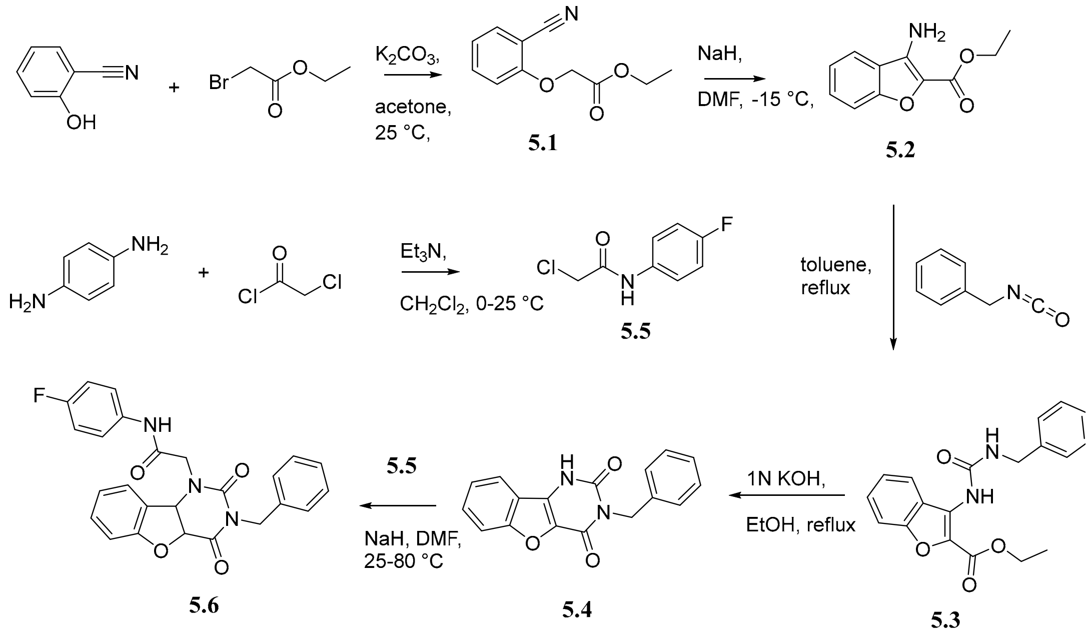

Figure S2. Synthetic scheme for compound series 5.

5.1. Ethyl 2-(2-Cyanophenoxy) Acetate

Potassium carbonate (10.5 g, 76.0 mmol) was added to a solution of 2-cyanophenol (6.00 g, 50.4 mmol) and ethyl bromoacetate (10.0 g, (59.9 mmol) in acetone (60 mL). The mixture was stirred overnight at room temperature and filtered. The filtrate was evaporated to an oil, and the crude product was recrystallized to give the title compound (9.21 g, 89%).

NMR (400 MHz, DMSO-d$_6$) δ (ppm): 7.76 (dd, *J* = 7.7, 1.7 Hz, 1H), (7.64, m, 1H), 7.17 (d, *J* = 8.6 Hz, 1H), 7.13 (dt, *J* = 7.6, 0.8 Hz, 1H), 5.02 (s, 2H), 4.18 (q, *J* = 7.1 Hz, 2H), 1.21 (t, J = 7.1 Hz, 3H); HPLC 100 area% (230 nm).

5.2. Ethyl 3-Aminobenzofuran-2-Carboxylate

A solution of ethyl 2-(2-cyanophenoxy)acetate (7.61 g, 37.1 mmol) in DMF (60 mL) was chilled to −15 °C (ice–salt bath) before the addition of NaH (60% dispersion, 2.27 g, 56.8 mmol). The mixture was stirred for another hour before being poured over ice–water. The mixture was extracted into EtOAc. Combined extracts were washed with saturated NaCl solution, dried ($MgSO_4$), filtered, and evaporated. The crude product was purified on a column of silica gel eluting with hexane/EtOAc (7:3). The appropriate fractions were evaporated to an oil, which solidified under high vacuum, gave the title compound (6.57, 86%).

^{1}H NMR (400 MHz, DMSO-d$_6$) δ (ppm): 7.94 (d, *J* = 7.9 Hz, 1H), 7.48 (d, *J* = 3.5 Hz, 2H), 7.25 (m, 1H), 6.34 (s, 2H), 4.29 (q, *J* = 7.1 Hz, 2H), 1.32 (t, *J* = 7.1 Hz, 3H); HPLC >98 area % (254 nm).

5.3. Ethyl 3-(3-Benzylureido) Benzofuran-2-Carboxylate

A solution of ethyl 3-aminobenzofuran-2-carboxylate (5.15 g, 25.1 mmol) in toluene (60 mL) was used to reflux in a 500 mL 3-neck flask fitted with a condenser and addition funnel. A solution of benzyl isocyanate (4.78 g, 3.9 mmol) in toluene (120 mL) was added dropwise over 2.5 hours. The addition funnel was removed, and the reaction mixture was concentrated using a Dean–Stark trap

inserted between the 3-neck flask and the condenser, until precipitation of the product began. After eight hours, the reaction mixture was allowed to cool to room temperature. The precipitate was filtered off, giving the title compound (4.64 g, 55%, HPLC 95.9 area %) as a white solid. The filtrate was treated as above with more benzyl isocyanate (3.55 g, 26.7 mmol) to give a second crop (1.11 g, total recovery 5.75 g, 68%).

^{1}H NMR (400 MHz, DMSO-d$_6$) δ (ppm): 8.77 (s, 1H), 8.15 (d, *J* = 8.2 Hz, 1H), 7.94 (t, *J* = 5.8 Hz, 1H), 7.61 (d, *J* = 8.4 Hz, 1H), 7.51 (m, 1H), 7.35 (m, 4H), 7.28 (m, 2H), 4.38 (m, 4H), 1.35 (t, *J* = 7.1 Hz, 3H).

5.4. 3-Benzylbenzofuro[3,2-d] Pyrimidine-2,4(1H,3H)-Dione

A mixture of ethyl 3-(3-benzylureido)benzofuran-2-carboxylate (10.30 g, 30.4 mmol) in EtOH (100 mL) and 1 N KOH solution (90 mL) was refluxed for 30 minutes. The mixture was gravity filtered into water (ca. 400 mL). The solution was stirred and acidified with 1 N HCl solution (75 mL). The mixture was diluted with water to 1 L and refrigerated overnight. The precipitate was filtered off and dried under high vacuum to give the title compound as a white solid (8.58 g, 96%); mp 327–328 °C.

^{1}H NMR (400 MHz, DMSO-d$_6$) δ (ppm): 8.03 (d, *J* = 7.9 Hz, 1H), 7.78 (d, *J* = 8.5 Hz, 1H), 7.66 (dt, *J* = 7.9, 1.2 Hz, 1H), 7.46 (t, *J* = 7.5 Hz, 1H), 7.32 (m, 4H), 7.25 (m, 1H), 5.10 (s, 2H); HPLC 98.8 area % (265 nm). Anal. Calcd for $C_{17}H_{12}N_2O_3 \cdot 0.2H_2O$: C, 69.01; H, 4.22; N, 9.47. Found: C, 68.66; H, 3.89; N, 9.40.

5.5. 2-Chloro-N-(4-Fluorophenyl) Acetamide

A solution of 4-fluoroaniline (580 mg, 5.22 mmol) and trimethylamine (d = 0.726, 0.9 mL, 6.46 mmol) in CH_2Cl_2 (10 mL) under Ar was chilled to 0 °C (ice–salt bath). Chloroacetyl chloride (d = 1.419, 0.5 mL, 6.28 mmol) was added dropwise by syringe. The cold bath was removed, and the mixture was stirred overnight at room temperature. The mixture was poured into water and extracted in CH_2Cl_2 (3 ×). Combined extracts were washed with saturated NaCl solution, dried ($MgSO_4$), filtered, and evaporated. The residue was recrystallized from toluene to give the title compound as white crystals (742 mg, 76%); mp 128–129 °C.

^{1}H NMR (400 MHz, DMSO-d$_6$) δ (ppm): 10.34 (s, 1H), 7.61 (dd, *J* = 9.2, 5.0 Hz, 2H), 7.17 (t, *J* = 8.9 Hz, 2H), 4.24 (s, 2H); HPLC 100 area % (254 nm). Anal. Calcd for C_8H_7ClFNO: C, 51.22; H, 3.76; N, 7.47; Cl, 18.90; F, 10.13. Found: C, 51.23; H, 3.64; N, 7.42; Cl, 18.74; F, 9.96.

5.6. 2-(3-Benzyl-2,4-Dioxo-3,4,4a,9b-Tetrahydrobenzofuro[3,2-d]Pyrimidin-1(2H)-yl)-N-(4-Fluorophenyl) Acetamide

Sodium hydride (60% dispersion, 57.3 ng, 1.43 mmol) was added to a suspension of 3-benzylbenzofuro[3,2-*d*]pyrimidine-2,4(1*H*,3*H*)-dione (295 mg, 1.01 mmol) in dry DMF (10 mL). The mixture was stirred for one hour. 2-Chloro-*N*-(4-fluorophenyl)acetamide (198 mg, 1.05 mmol) was added (solids dissolved), and the solution was stirred at 80 °C for two hours. The resulting precipitate was filtered off and dried under high vacuum (P_2O_5). The crude product (433 mg, 97% recovery, mp 257–259 °C) was recrystallized from EtOH to give the title compound as a white solid (334 mg, 75%), mp 260–262 °C.

^{1}H NMR (400 MHz, DMSO-d$_6$) δ (ppm): 10.55 (s, 1H), 8.03 (d, *J* = 8.1 Hz, 1H), 7.86 (d, *J* = 8.5 Hz, 1H), 7.70 (td, *J* = 7.8, 1.0 Hz, 1H), 7.56 (dd, *J* = 9.2, 5.0 Hz, 2H), 7.46 (t, *J* = 7.4 Hz, 1H), 7.33 (m, 4H), 7.26 (m, 1H), 7.16 (t, *J* = 8.9 Hz, 2H), 5.17 (s, 4H); ESI MS *m/z* = 444.1 ([M + 1]$^+$) ; HPLC 100 area % (230 nm). Anal. Calcd for $C_{25}H_{18}FN_3O_4$: C, 67.72; H, 4.09; N, 9.48. Found: C, 67.68; H, 3.97; N, 9.44.

Compounds Series 6:

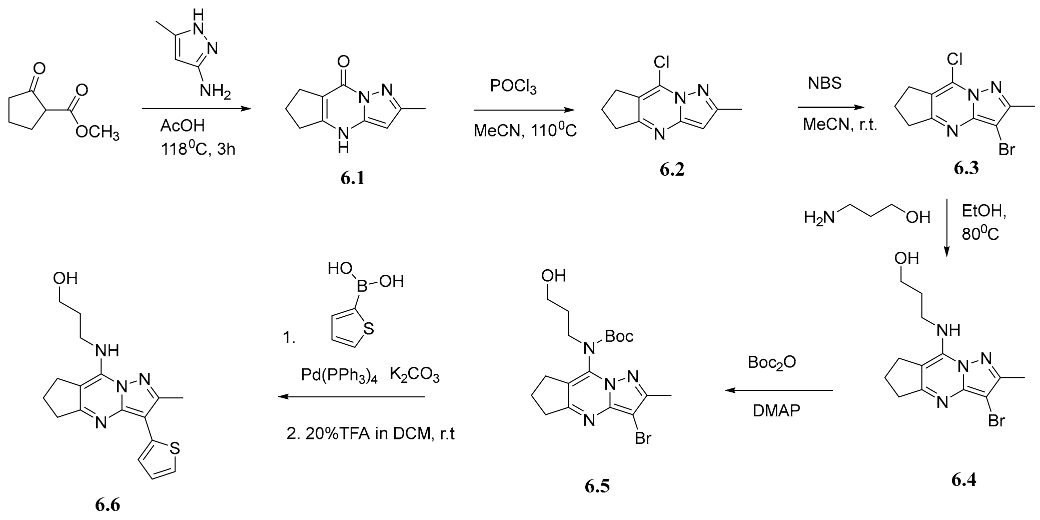

Figure S3. Synthetic scheme for compound series 6.

6.1. 11-Methyl-1,8,12-Triazatricyclo[7.3.0.0^{3,7}]Dodeca-3(7),9,11-Trien-2-One

Methyl 2-oxocyclopentanecarboxylate 8.0 g (56.63 mmol) and 3-Amino-5-methylpyrazole 5.0 g (51.68 mmol) were stirred under reflux in 50 mL of acetic acid. The reaction was monitored by TLC (10% MeOH in DCM). After 3 h of reaction was complete, the mixture was cooled down and solvent removed by rotovap. Residue diluted with EtOAc, filtered, and dried in vacuum. Obtained 9.2 g (94%) of product **6.1**.

6.2. 2-Chloro-11-Methyl-1,8,12-Triazatricyclo[7.3.0.0^{3,7}] Dodeca-2,7,9,11-Tetraene

A total of 8.0 g (42.3 mmol) of compound **6.1** in 30 mL $POCl_3$ was heated at 110 °C for 4 h. The reaction progress was monitored by TLC (10% MeOH in DCM). A small aliquot was taken from the reaction mixture poured into water, the formed precipitate was centrifuged, dissolved in EtOAc, and the solution placed on TLC. After the reaction was completed, the reaction mixture was poured into crushed ice (500 g). The formed precipitate was collected, washed with water, and dried in vacuum. Obtained 8.5 g (97.3%) of product **6.2**, which was used in the next step without any further purification.

6.3. 10-Bromo-2-Chloro-11-Methyl-1,8,12-Triazatricyclo [7.3.0.0^{3,7}] dodeca-2,7,9,11-Tetraene

A total of 8.0 g (38.5 mmol) of compound **6.2** and 7.5 g (42.1 mmol) of *N*-bromosuccinimide were stirred in 160 mL of MeCN at r.t. under nitrogen atmosphere. Reaction progress was monitored by TLC (30% EtOAc in hexane). After 3 h, the reaction was complete and the solvent was removed by rotovap. To the residue, saturated aq NaHCO3 was added and the mixture extracted with EtOAc. Organic layers were combined, washed with water and saline. The solvent was removed, and the residue was dried under vacuum. Obtained 7.5 g (68.2%) of compound **6.3**, which was used in the next step without purification.

6.4. 3-({10-Bromo-11-Methyl-1,8,12-Triazatricyclo[7.3.0.0^{3,7}]Dodeca-2,7,9,11-Tetraen-2-Yl}amino) Propan-1-Ol

A total of 5.3 g (70.7 mmol) of 3-amino-1-propanol, 7.0 g (24.4 mmol) of compound **6.3** were stirred under reflux in 50 mL of EtOH. The reaction progress was monitored by TLC (50% EtOAc in hexane). The reaction mixture was refluxed overnight (12 h) and the solvent removed by rotovap. Water was added to the residue and the product extracted with CH_2Cl_2. Organic layers were combined, washed with saline, and dried with Na_2SO_4. The solvent was removed and the residue

purified by column chromatography (eluent 50% EtOAc in hexane). Obtained 5.1 g (64.3%) of the title compound.

6.5. Tert-Butyl N-{10-Bromo-11-Methyl-1,8,12-Triazatricyclo[7.3.0.0^{3},^{7}] Dodeca-2,7,9,11-Tetraen-2-Yl}-N-(3-Hydroxypropyl) Carbamate

To a solution of compound **6.4,** 5.0 g (15.4 mmol) in dry dioxane (30 mL) and 2.0 g (16.4 mmol) N,N-dimethyl-4-aminopyridine was added, followed by 6.7 g (30.8 mmol) of Boc_2O. The reaction mixture was stirred at r.t. for 2 h. Reaction progress was monitored by TLC (30% EtOAc in Hexane). Solvent was removed by rotovap. To the residue, water was added and extracted with DCM. Organic layers were combined, washed with sat. aq. $NaHCO_3$, brine, and dried with Na_2SO_4. The solution was filtrated, the solvent removed, and residue purified by column chromatography (eluent 30% EtOAc in Hexane). Obtained 5.3 g (81.0 %) of product **6.5**.

6.6. 3-{[11-Methyl-10-(Thiophen-2-Yl)-1,8,12-Triazatricyclo [7.3.0.0^{3},^{7}] Dodeca-2,7,9,11-Tetraen-2-Yl] Amino}propan-1-Ol

A sample of 4.0 g (9.4 mmol) of compound **6.5** was dissolved in 50 mL of toluene : EtOH = 9:1 mixture. To this solution, 2.4 g (18.8 mmol) of 2-thiophen boronic acid and 3.3 g (23.9 mmol) K_2CO_3 were added and the mixture stirred for 30 min followed by 0.87 g (0.75 mmol) of $Pd(PPh_3)_4$. The resulting reaction mixture was stirred under reflux overnight. The reaction progress was monitored by TLC (10% MeOH in DCM). Solvent removed by rotovap and to the residue sat. aq. $NaHCO_3$ was added. The mixture was extracted with DCM. Organic layers were combined, washed with brine, and dried with Na_2SO_4. The solvent was removed and residue purified by flash chromatography (eluent 10% MeOH in DCM). Obtained 1.7 g (39.5%). A total of 1.5 g of Boc-protected amine was stirred at r.t in 20 mL 20%TFA/DCM. The reaction was monitored by TLC (10% MeOH in DCM). The reaction was complete after 2 h and the reaction mixture was poured into an aqueous solution of $NaHCO_3$ and extracted with DCM. Organic layers were combined, washed with saline, and dried over Na_2SO_4. The solvent was removed in rotovap and the residue purified by column chromatography (10% MeOH in DCM). Obtained 0.9 g (78.3%) of title compound **6.6**.

^{1}H NMR (300 MHz, $CDCl_3$) δ (ppm): 7.73 (dd, 1H, J = 1.2 Hz, J = 5.1 Hz), 7.62 (dd, 1H, J = 1.2 Hz, J = 3Hz), 7.41 (dd, 1H, J = 2.7 Hz, J = 4.8 Hz), 6.56 (broad t, 1H), 3.94–3.79 (m, 4H), 3.23–3.16 (m, 2H), 3.02–2.95 (m, 2H), 2.63 (s, 3H), 2.23–2.12 (m, 2H), 2.07–1.93 (m, 2H). ESI MS m/z = 330.7 ([M + H]$^{+}$).

Compounds Series 7:

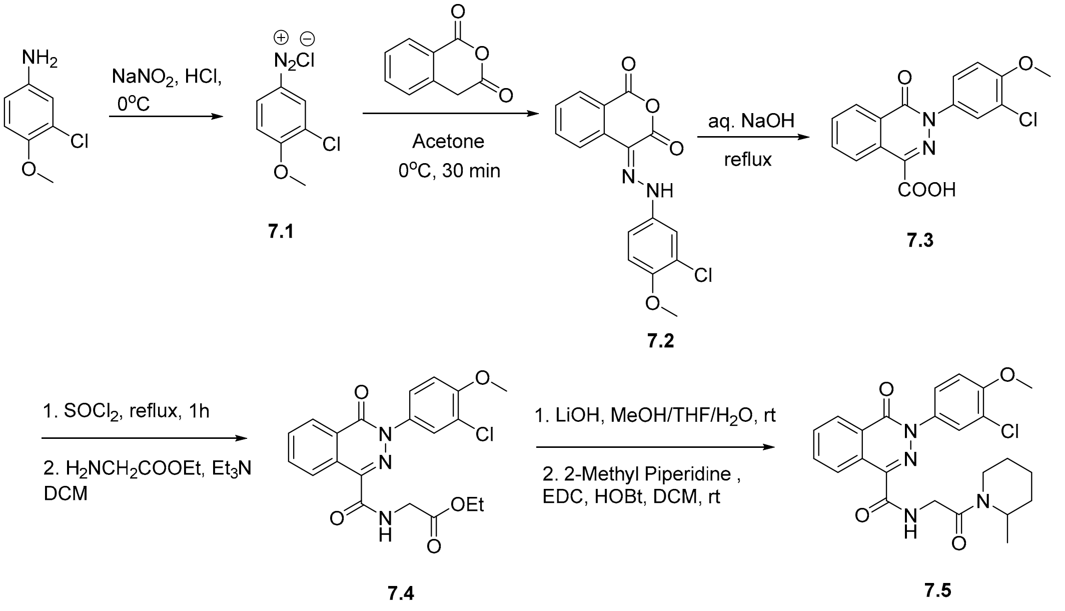

Figure S4. Synthetic scheme for compound series 7

7.2. (4Z)-4-[2-(3-Chloro-4-Methoxyphenyl) Hydrazin-1-Ylidene]-3,4-Dihydro-1H-2-Benzopyran-1,3-Dione

To a solution of anhydride (1.62 g, 10 mmol) in acetone (80 mL) at 0 °C was added a cold solution of 3-chloro-4-methoxydiazonium chloride (**7.1**) [prepared by diazotizing 3-chloro-4-methoxyaniline (1.58 g, 10 mmol) in 6 N hydrochloric acid (8 mL) with sodium nitrite (0.69 g, 10 mmol) in water (10 mL)] with constant stirring. Compound **7.2** separated within a short time. After stirring for additional 0.5 h, water (400 ml) was added and the product filtered, washed with water, dried, and crystallized from xylene giving bright yellow needles, 2.3 g (72%).

7.3. (3-Chloro-4-Methoxyphenyl)-4-Oxo-3,4-Dihydrophthalazine-1-Carboxylic Acid

Compound **7.2** (3.3 g, 10 mmol) was boiled with 5% aqueous sodium hydroxide (50 mL) for 15–20 min when a clear solution was obtained. The solution was cooled and carefully acidified with dilute hydrochloric acid to obtain the crude product (**7.3**). The crude product was separated, dissolved in 2% sodium hydrogen carbonate (70 mL) solution and filtered. The filtrate on acidification with dilute hydrochloric acid gives a pale yellow color (**7.3**), which was filtered, washed with water, dried, and crystallized from glacial acetic acid to give fine white needles, yielding 3.0 g (93%).

7.4. Ethyl 2-{[3-(3-Chloro-4-Methoxyphenyl)-4-Oxo-3,4-Dihydrophthalazin-1-Yl] Formamido} Acetate

Acid 7.3 (1 g, 3 mmol) and thionyl chloride (5 mL) was reflexed for 1 h. The reaction mixture became clear within 10 min. After that, the thionyl chloride was completely removed under vacuum and dried. The crude product was used next without any purification. To a suspension of glycine ethyl ester hydrochloride (0.54 g, 3.9 mmol) in DCM (20 mL) was added triethylamine (0.45 g, 4.5 mmol). The resulting solution was then cooled to 0° C and a solution of acid chloride in DCM (10 mL) was added dropwise. The reaction mixture was stirred at 0 °C for 2 hours and then at room temperature for 18 hours. The resulting solution was diluted with DCM (50 mL) and washed with 10 % HCl (2 x 25 mL). The organic phase was separated, dried (MgSO4), and concentrated in vacuo to afford the title compound **7.4**. Further purification with 3% DCM in MeOH will give pure ester (**7.4**) with a yield of 1.0 g (80%) as a white solid.

7.5. 3-(3-Chloro-4-Methoxyphenyl)-N-[2-(2-Methylpiperidin-1-Yl)-2-Oxoethyl]-4-Oxo-3,4-Dihydrophthalazine-1-Carboxamide

LiOH (0.103 g, 4.30 mmol) was added to a stirred solution of ester **7.4** (0.6 g, 1.44 mmol) in THF:MeOH:H_2O (3:1:1,10 mL) and the resulting mixture was stirred at room temperature for five hours. The MeOH and THF were then evaporated and the residue was diluted with water (2 mL), acidified with 20% aq HCl, and extracted with EtOAc. The organic layer was washed with saturated brine solution, dried over Na_2SO_4, and concentrated under reduced pressure to afford acid in a quantitative yield as a white solid. The resulting acid was dissolved in dry CH_2Cl_2 and sequentially treated with HOBt (0.29 g, 2.16 mmol) and EDCI (0.413 g, 2.16 mmol) at 0 °C under a nitrogen atmosphere. After 10 minutes, 2-methylpiperidine (0.214 g, 2.16 mmol) was followed by DIPEA (0.28 g, 2.16 mmol). The reaction mixture was allowed to reach room temperature and stirred further for 10 h under a nitrogen atmosphere. After that, the reaction mixture was diluted with $CHCl_3$, washed with 1 N HCl, water, and aq. saturated $NaHCO_3$ solution and aq. NaCl solution. The organic layer was dried (Na_2SO_4) and evaporated to give the crude, which was purified by column chromatography with 5% DCM in MeOH, affording **7.5** in a yield of 0.473 g (70%) as a white solid.

^{1}H NMR (300 Hz, $CDCl_3$) δ (ppm): 9.8 (d, 1H, *J* = 8.1 Hz), 8.51 (dd, 1H, *J* = 1.5 Hz, *J* = 9.0 Hz), 8.15 (t, 1H, 1.5 Hz), 7.91–7.78 (m, 2H), 7.73 (d, 1H, *J* = 2.7 Hz), 7.54 (dd, 1H, *J* = 2.7 Hz, *J* = 9.0 Hz), 7.4 (d, 1H, *J* = 8.7 Hz), 4.88 (bs, 0.5H), 4.50–4.00 (m, 2.5H), 3.97 (s, 3H), 3.53 (d, 0.5H, *J* = 10.8 Hz), 3.19 (t, 0.5H, *J* = 12.9 Hz), 2.73 (t, 0.5H, *J* = 12.9 Hz), 1.80–1.60 (m, 4H), 1.35–1.15 (m, 3H), ESI MS m/z = 469.4 ([M + H]$^+$).

Compounds Series 8:

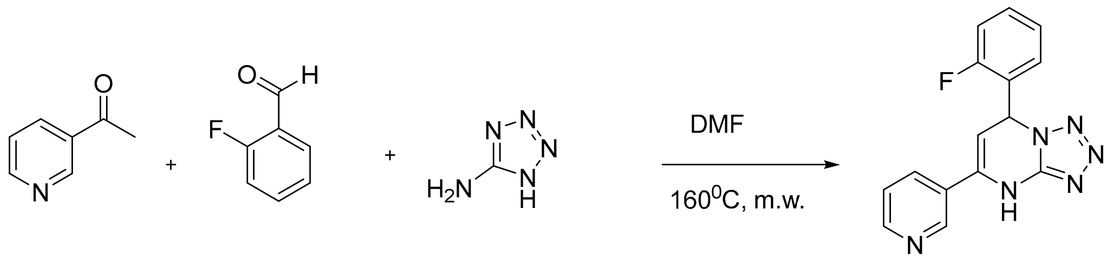

8.1

Figure S5. **Synthetic scheme for compound series 8.**

8.1. 3-[7-(2-Fluorophenyl)-4H,7H-[1,2,3,4] Tetrazolo[1,5-A]pyrimidin-5-Yl]pyridine

A sample of 1 mmol of 3-acetylpyridine, 2-fluorobenzaldehyde and 5-aminotetrazole was placed into a microwave tube to which 1 mL of DMF was added. The tube was flushed with nitrogen, sealed, and heated under microwave irradiation at 160 °C for 40 min. The reaction progress was monitored with TLC (10% MeOH in DCM). The formed product was purified by column chromatography (10% MeOH in DCM).

^{1}H NMR (300 MHz, $CDCl_3$) δ (ppm): 8.95 (d, 1H, J = 2.4 Hz), 8.70 (dd, 1H, J = 1.5 Hz, J = 4.8 Hz), 8.20 (dt, 1H, J = 1.8 Hz, J = 5.7 Hz), 7.50 (dd, 1H, J = 4.2 Hz, J = 8Hz), 7.44–7.32 (m, 1H), 7.27–7.10 (m, 3H), 6.84 (d, 1H, J = 3.6 Hz), 5.32 (m, 1H). ESI MS m/z = 295.2 ($[M + H]^+$)

Compounds Series 10:

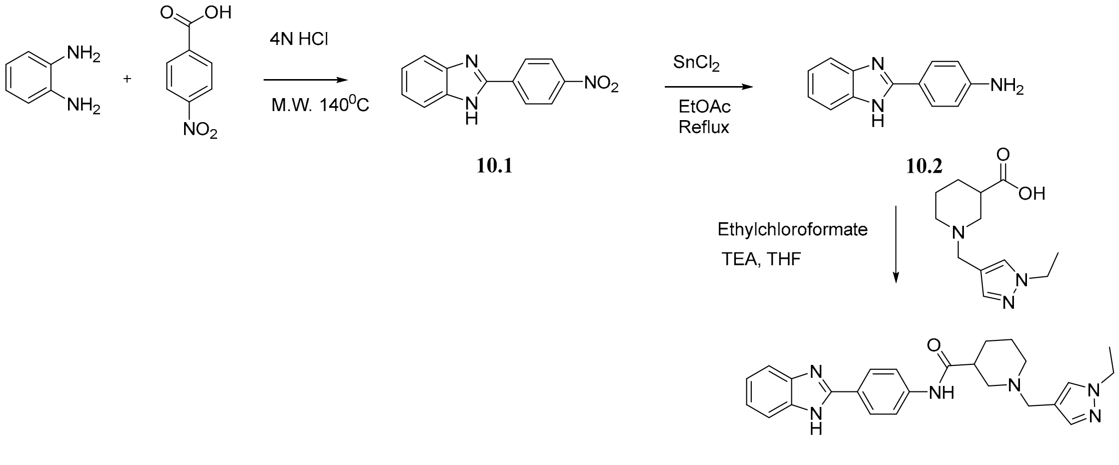

10.3

Figure S6. **Synthetic scheme for compound series 10.**

10.1. 2-(4-Nitrophenyl)-1H-1,3-Benzodiazole

A total of 2.0 g (18.5 mmol) of o-phenylenediamine, 3.09 g (18.5 mmol) of p-nitrobenzoic acid, and two drops 4 N HCl were mixed and subjected to microwave irradiation at 140 °C for 3 h. The reaction progress was monitored by TLC (40% EtOAc in hexane). Water was added to the mixture and pH was adjusted to 5–6 with 10% NaOH. The product was extracted with DCM. The solvent was removed and the crude product recrystallized from ethanol–water, yielding 4.2 g (95%) of pure compound **10.1**

10.2. 4-(1H-1,3-Benzodiazol-2-Yl) Aniline

A total of 800 mg (3.37 mmol) of nitro-compound **10.1** was dissolved in 50 mL of EtOAc. To this solution, 3.79 g (16.87 mmol) of tin(II)chloride monohydrate was added. The reaction mixture was

stirred under reflux for 6 h. Next, it was cooled down and poured into aq. $NaHCO_3$ and extracted with EtOAc. The organic layers were combined, washed with brine, and dried over Na_2SO_4. The solvent was removed by rotovap. The crude product **10.2** was used in the next step without further purification.

10.3. N-[4-(1H-1,3-Benzodiazol-2-Yl)Phenyl]-1-[(1-Ethyl-1H-Pyrazol-4-Yl) Methyl]piperidine-3-Carboxamide

A total of 50.0 mg (0.21 mmol) of 1-[(1-ethyl-1H-pyrazol-4-yl)methyl]piperidine-3-carboxylic acid and 25.1 mg (0.23 mmol) of ethyl chloroformate in 3 mL of dry THF were stirred at r.t. for 5 min. The mixture was cooled down to 0 °C and 52.9 mg (0.25 mmol) of compound **10.2** and 53.3 mg (0.53 mmol) of trimethylamine were added. The reaction mixture was stirred at r.t. for 3 h. The reaction progress was monitored by TLC (10% MeOH in DCM). The mixture was poured into aq. $NaHCO_3$ and extracted with EtOAc. Organic layers collected washed with brine and dried over Na_2SO_4. The solution was filtrated and the solvent removed. The residue was purified by column chromatography. Obtained 61 mg (68%) of pure product **10.3**.

^1H NMR (300 MHz, CD_3OD) δ (ppm): 8.06 (d, 2H), 7.76 (d, 2H), 7.64 (s, 1H), 7.60 (bs, 2H), 7.48 (s, 1H), 7.30–7.22 (m, 2H), 4.16 (q, 2H), 3.54 (s, 2H), 2.92 (m, 2H), 2.66 (m, 1H), 2.36 (t, 1H), 2.20 (t, 1H), 2.00-1.52 (m, 3H), 1.44 (t, 3H). ESI MS m/z = 429.6 ($[M + H]^+$), m/z = 857.6 ($[2M + H]^+$).

Compounds Series 11:

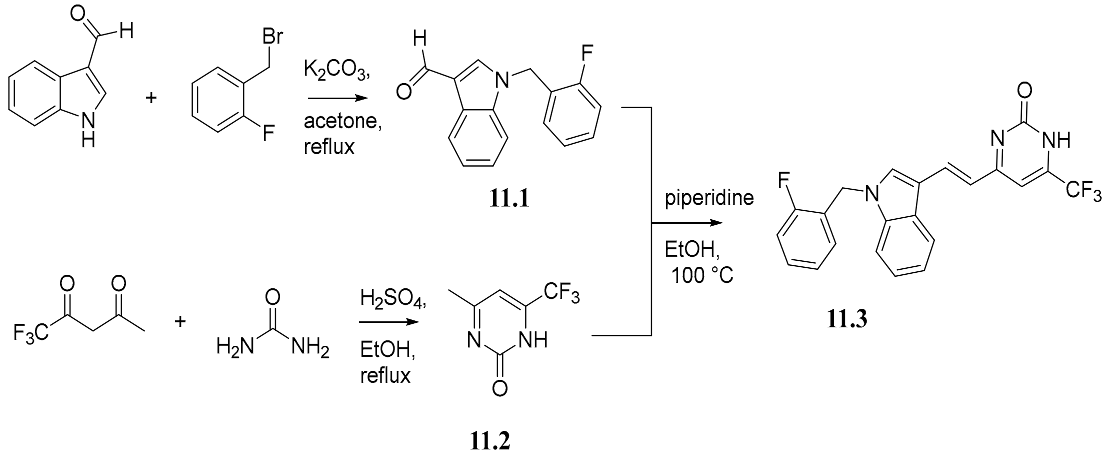

Figure S7. Synthetic scheme for compound series 11.

11.1. 1-(2-Fluorobenzyl)-1H-Indole-3-Carbaldehyde

Potassium carbonate (5.28 g, 4.00 mmol) was added to a solution of indole-3-carboxaldehyde (1.00 g, 6.89 mmol) in acetone (40 mL). The mixture was stirred for one hour at room temperature. A solution of 2-fluorobenzyl bromide (1.37 g, 7.23 mmol) in acetone (10 mL) was added and the mixture was refluxed for 3.5 hours until the reaction was complete by HPLC. The cooled mixture was filtered. The filtrate was evaporated, dissolved in CH_2Cl_2, and washed with water. The organic layer was dried ($MgSO_4$), filtered, and evaporated. The crude product was purified on a silica gel column eluting with gradient from neat hexane to hexane/EtOAc (3:2), to give the title compound as a yellow solid (1.50 g, 86%).

^1H NMR (400 MHz, $CDCl_3$) δ (ppm): 10.02 (s, 1H), 8.35–8.31 (m, 1H), 7.76 (s, 1H), 7.42–7.37 (m, 1H),), 7.36–7.29. (m, 3H), (7.19–7.10, m, 1H), 7.10–7.03 (m, 2H), 5.40 (s, 2H); HPLC 97.5 area % (254 nm).

11.2. 4-Methyl-6-(Trifluoromethyl) Pyrimidin-2(1H)-One

Urea (937 mg, 15.6 mmol) was added to a stirred solution of 1,1,1-trifluoropentane-2,4-dione (2.00 g, 12.98 mmol) and H_2SO_4 (1 mL) in EtOH (50 mL). The mixture was refluxed under Ar for 48

hours. The reaction mixture was neutralized with saturated $NaHCO_3$, extracted with diethyl ether, and dried over $MgSO_4$. The solvent was removed under reduced pressure, and the title compound was recrystallized from ethanol as white crystals (mp 178–180 °C, lit.[1] mp 188–190 °C).

^1H NMR (400 MHz, $CDCl_3$) δ (ppm): 6.61 (s, 1H), 4.2 (s, 1H), 2.49 (s, 3H).

11.3. (E)-4-(2-(1-(2-Fluorobenzyl)-1H-Indol-3-Yl) Vinyl)-6-(Trifluoromethyl) Pyrimidin-2(1H)-One

Piperidine (nine drops, 0.0561 mmol) was added to a solution of 1-(2-fluorobenzyl)-indole-3-carbaldehyde (2.14 mg, 0.842 mmol) and 4-methyl-6-trifluromethyl-2(1*H*)-pyrimidinone (100 mg, 0.561 mmol) in dry EtOH (15 mL). The mixture was stirred at 100 °C for 3.5 hours in a sealed tube. The reaction mixture was evaporated to an orange solid (300 mg). The crude product was purified on a column of silica gel eluting with a gradient from hexane/EtOC (85:15) to neat EtOAc. The title compound was isolated as a solid (213 mg, 92%), mp 276–278 °C.

^1H NMR (400 MHz, DMSO-d_6) δ (ppm): 12.36◎(s, 1H), 8.29 (d, *J* = 16.3 Hz, 1H), 8.08–7.99 (m, 1H), 7.96 (s, 1H), 7.65–7.56 (m, 1H), 7.35 (tdd, *J* = 7.6, 5.3, 1.9 Hz, 1H), 7.31–7.10 (m, 6H), 7.01 (d, *J* = 16.3 Hz, 1H), 5.57 (s, 2H); HPLC 100% area (254 nm). Anal. Calcd for $C_{22}H_{15}F_4N_3O{\cdot}0.6H_2O$: C, 62.29; H, 3.85; N, 9.91. Found: C, 62.11; H, 3.99; N, 9.52.

References

1. Sloop, J.C.; Bumgardner, C.L.; Loehle, W.D. Synthesis of fluorinated heterocycles. *J. Fluor. Chem.* **2002**, *118*, 135–147.

© 2020 by the authors. Submitted for possible open access publication under the terms and conditions of the Creative Commons Attribution (CC BY) license (http://creativecommons.org/licenses/by/4.0/).
